# Association between Sleep Apnea Hypopnea Syndrome and the Risk of Atrial Fibrillation: A Meta-Analysis of Cohort Study

**DOI:** 10.1155/2018/5215868

**Published:** 2018-01-18

**Authors:** Enfa Zhao, Shimin Chen, Yajuan Du, Yushun Zhang

**Affiliations:** ^1^Department of Structural Heart Disease, The First Affiliated Hospital of Xi'an Jiaotong University, Xi'an, Shaanxi, China; ^2^Department of Gastroenterology, The First Affiliated Hospital of Shaanxi University of Chinese Medicine, Xianyang, Shaanxi, China

## Abstract

Numerous reports have been done to seek the relationship between sleep apnea hypopnea syndrome (SAHS) and the risk of atrial fibrillation (AF). However, definite conclusion has not yet been fully established. We examined whether SAHS increases AF incidence in common population and summarized all existing studies in a meta-analysis. We summarized the current studies by searching related database for potential papers of the association between SAHS and the risk of AF. Studies that reported original data or relative risks (RRs) with 95% confidence intervals (CIs) for the associations were included. Sensitivity analyses were performed by omitting each study iteratively and publication bias was detected by Begg's tests. Eight eligible studies met the inclusion criteria. Fixed effects meta-analysis showed that SAHS increased AF risk in the common population (RR = 1.70, 95% CI: 1.53–1.89, *P* = 0.002, *I*^2^ = 69.2%). There was a significant association between mild SAHS and the risk of AF (RR = 1.52, 95% CI: 1.28–1.79, *P* = 0.01, *I*^2^ = 78.4%), moderate SAHS (RR = 1.88, 95% CI: 1.55–2.27, *P* = 0.017, *I*^2^ = 75.6%), and severe SAHS (RR = 2.16, 95% CI: 1.78–2.62, *P* < 0.001, *I*^2^ = 91.0%). The results suggest that sleep apnea hypopnea syndrome could increase the risk of AF, and the higher the severity of SAHS, the higher risk of atrial fibrillation.

## 1. Introduction

Atrial fibrillation (AF) is the most common sustained cardiac arrhythmia encountered in clinical practice, affecting approximately 1% to 4% of the general population [1]. AF is associated with marked morbidity and increased mortality [1, 2]. It is reported that AF has also been associated with several disease processes such as hypertension, diabetes mellitus, heart failure, myocardial infarction, and valvular heart disease [3]. In recent years, there has been a growing interest about the link between sleep apnea hypopnea syndrome and atrial fibrillation (AF) [4]. Sleep apnea hypopnea syndrome (SAHS) is characterized by recurrent partial or complete collapse of upper airway during sleep and is estimated to affect approximately 5% of adult women and 14% of men [5]. It is reported that SAHS is common in the general population, especially among patients with established cardiovascular disease, including obesity, sedentary life, and increasing age [6]. Several studies have reported a higher prevalence of AF in patients with sleep apnea as compared with those without SAHS [7–9]. However, the conclusions of several observational studies are inconsistent with regard to AF risk. Six studies [3, 10–14] confirmed the link between SAHS and AF, while two studies [15, 16] failed to show any significant association between SAHS and AF.

Therefore, this study aims to conduct a meta-analysis by combining the results from all available cohort studies to examine whether SAHS increases AF incidence in common population and evaluate the risk of AF for mild SAHS (obstructive apnea hypopnea index, 5%–14.9%), moderate SAHS (obstructive apnea hypopnea index, 15%–29.9%), and severe SAHS (obstructive apnea hypopnea index, *⩾*30), respectively.

## 2. Materials and Methods

### 2.1. Literature Search

**Figure 1:**
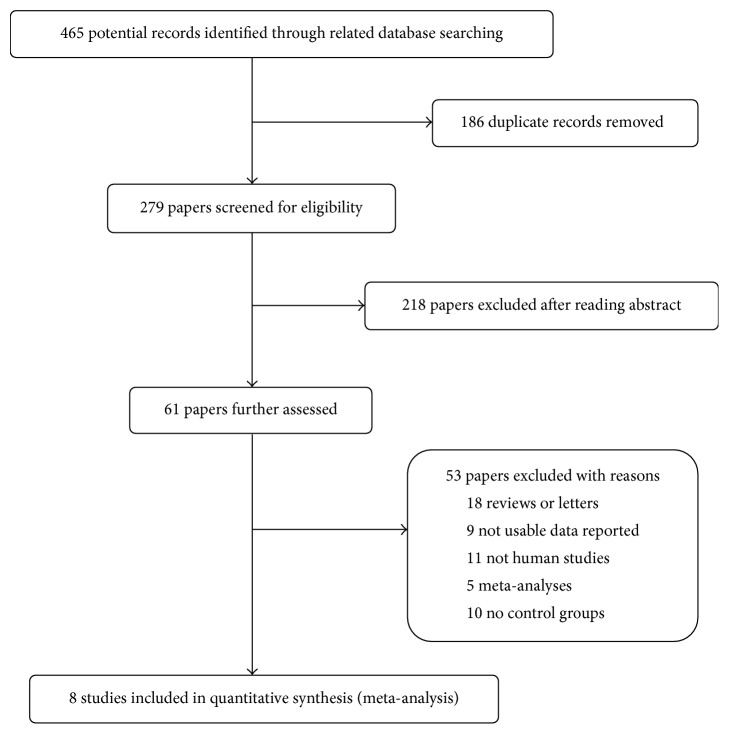
The detailed procedures for the literature search.

The meta-analysis was performed based on the preferred reporting items for systematic reviews and meta-analysis (PRISMA) guidelines [17]. Two investigators independently performed a systematic literature search in any language on July 1, 2017, in EMBASE, PubMed, the Cochrane Library, and the Web of Science without limiting the publication date range. We used the following terms to identify relevant citations: (sleep apnea hypopnea syndromes or sleep apnea syndromes or sleep-disordered breathing or obstructive sleep apnea or obstructive sleep apnea hypopnea syndrome or central sleep apnea or central sleep apnea syndromes or mixed sleep apnea or obstructive apnea hypopnea index or SAHS or OSA or OAHI) and (atrial fibrillation or AF). We also reviewed the reference lists of identified articles to search additional eligible studies. The detailed procedures for the literature search are shown in Figure 1.

### 2.2. Inclusion and Exclusion Criteria

The following inclusive selection criteria were applied: (1) the study design was based on retrospective or perspective cohort studies; (2) the study evaluated the association between sleep apnea hypopnea syndrome and the risk of atrial fibrillation; (3) a study reporting AF number in SAHS group and AF number in the control group or the relative risks (RRs) with 95% confidence intervals (95% CIs) was included; (4) one of the end points of interest were incident HF; (5) study must contain reference group. Only studies that provided a percentage of the incidence of AF or did not provide available data to allow calculation of the effect estimates were excluded.

### 2.3. Data Extraction

The following information was recorded by two authors independently for each study using a standardized form: first author, country in which the study was performed, year of publication, study design, follow-up duration, number in case and control groups, and SAHS diagnostic methods. Two investigators independently performed a quality assessment using the 9-star Newcastle-Ottawa Scale (NOS) [18], and the NOS score was verified by a third investigator. We considered papers with NOS scores of 1–3, 4–6, and 7–9 for low, intermediate, and high-quality studies, respectively.

### 2.4. Statistical Analysis

We performed a meta-analysis to examine the relationship between SAHS and AF risk. Relative risk (RR) with 95% confidence interval (CIs) was used to estimate the effect sizes. A fixed effects model was used to estimate the pooled RR with 95% CIs if there was no significant heterogeneity (*P* > 0.05 and *I*^2^ < 50%); otherwise, a random effect model was used. Heterogeneity was considered as either a *P* value < 0.05 or *I*^2^ > 25% [19]. Sensitivity analysis was performed by omitting one study at a time to find potential outliers. We used Begg's test (rank correlation method) [20] to evaluate potential publication bias, and a *P* value of <0.1 was considered as significant statistical publication bias. Stata (Version 11.0; StataCorp, College Station, TX) was used for all statistical analyses.

## 3. Results

### 3.1. Characteristics of the Included Studies

Detailed studies retrieval procedures and selection result are shown in Table 1. A total of eight studies [3, 10–16] were included in this meta-analysis, involving 603532 non-SAHS and 14799 SAHS cases. Year of publication ranged from 2007 to 2017. The minimum mean age was 38.9 years and the maximum mean age was 75.0 years. Patients were followed up from 2 years and 4 months to mean 9.2 years. Of the 8 included studies, six were performed in the USA [3, 10, 11, 14–16], one in Australia [13], and one in China [12]. Three of eight studies reported the severity of SAHS [3, 13, 15]. The quality of studies evaluated by the NOS is shown in Table 1. Quality assessment showed that the mean NOS score was 7.25, indicating that the methodological quality was generally good. Table 1 includes the general characteristics of the eligible studies.

### 3.2. Meta-Analysis Results

**Figure 2:**
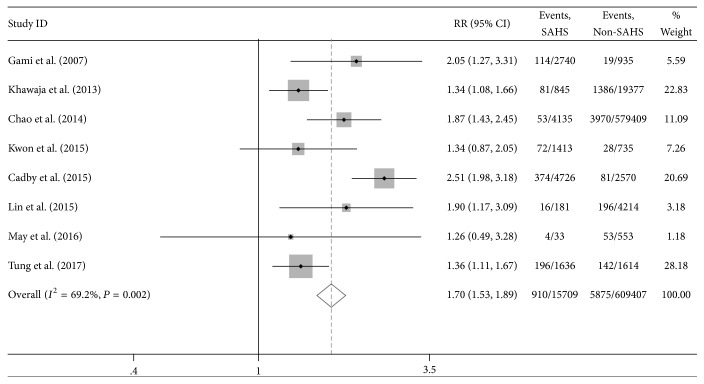
Relative risks (RRs) for the association between sleep apnea hypopnea syndrome and atrial fibrillation in 8 studies.

Meta-analysis was conducted to ascertain the potential relationship between sleep apnea hypopnea syndrome and the risk of atrial fibrillation among eight studies. As shown in Figure 2, the pooled results indicated that SAHS increased the risk of AF significantly compared to the common population with an increased risk of AF (RR = 1.70, 95% CI: 1.53–1.89) with noticeable heterogeneity (*I*^2^ = 69.2%, *P* = 0.002). We extracted variables that variables that affect research results. Then we further performed meta-analysis to pool the results in studies that adjusted for confounders. After adjustment for the confounders, there was still a positive association between SAHS and the risk of AF with an RR of 1.40 (95% CI: 1.12–1.74, *P* < 0.001).

**Figure 3:**
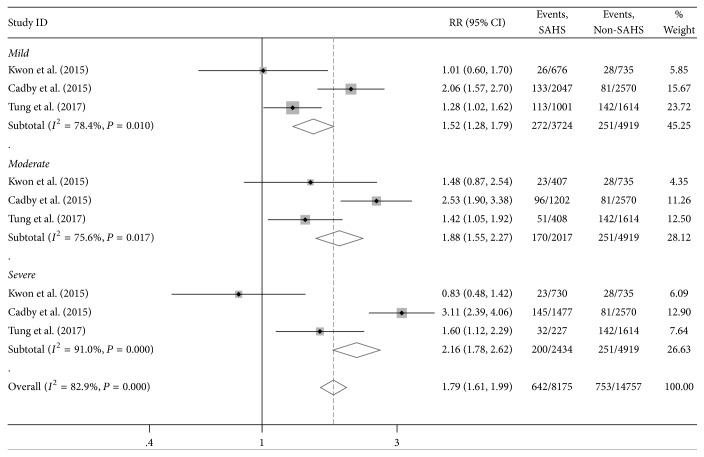
Dose-response relationships between sleep apnea hypopnea syndrome severity and atrial fibrillation risk in three studies.

Three studies reported the severity of SAHS. The obstructive apnea hypopnea (OAHI) was the main index of SAHS severity, defined as the number of obstructive apneas plus hypopneas per hour of sleep. Those with an OAHI of <5% were considered without SAHS (normal). An OAHI of 5%–14.9%, 15%–29.9%, and *⩾*30 was regarded as mild, moderate, and severe OAHI, respectively, based on clinically accepted cut points [3, 13, 15]. There is a dose-response relationship between SAHS severity and rates of incident AF. The dose-response analysis results (Figure 3) indicated that there was a significant association between mild SAHS and the risk of AF (RR = 1.52, 95% CI: 1.28–1.79, *P* = 0.01, *I*^2^ = 78.4%), moderate SAHS (RR = 1.88, 95% CI: 1.55–2.27, *P* = 0.017, *I*^2^ = 75.6%), and severe SAHS (RR = 2.16, 95% CI: 1.78–2.62, *P* < 0.001, *I*^2^ = 91.0%). The results also suggested that the higher the severity of SAHS, the higher the risk of atrial fibrillation.

### 3.3. Sensitivity Analysis and Metaregression

Sensitivity analysis was performed by subgroup analyses, which were conducted to handle the clinical heterogeneity across studies according to NOS score and countries. According to the countries where studies were performed, the studies were divided into three subgroups: USA (6 studies), China (one study), and Australia (one study). Meta-analysis of studies performed in USA showed that SAHS was associated with an increased risk of AF (RR = 1.42, 95% CI: 1.25–1.62) without heterogeneity (*I*^2^ = 0, *P* = 0.522). According to the NOS score of each study, the studies were divided into three subgroups. Three studies had been scored NOS of 8, four studies scored NOS of 7, and one study scored NOS of 6. Meta-analysis of studies with NOS score of 8 showed that AF risk increased due to SAHS (RR = 2.17, 95% CI = 1.77–2.67, *I*^2^ = 28.4%, *P* = 0.247). Studies with NOS score of 7 yielded an RR of 1.35 (95% CI = 1.17–1.55, *I*^2^ = 0, *P* = 0.999). Since noticeable heterogeneity was found to be present, metaregression analysis was performed to explore potential sources of heterogeneity. Out of all of the parameters, patients' age, follow-up duration, and countries where studies were performed were significant sources of heterogeneity (*P* = 0.045, 0.018, and 0.015, resp.). None of the publication year, gender, sample size, NOS score, and SAHS diagnosis was source of heterogeneity (*P* > 0.05). The meta-regression analysis results are shown in Table 2.

### 3.4. Publication Bias

**Figure 4:**
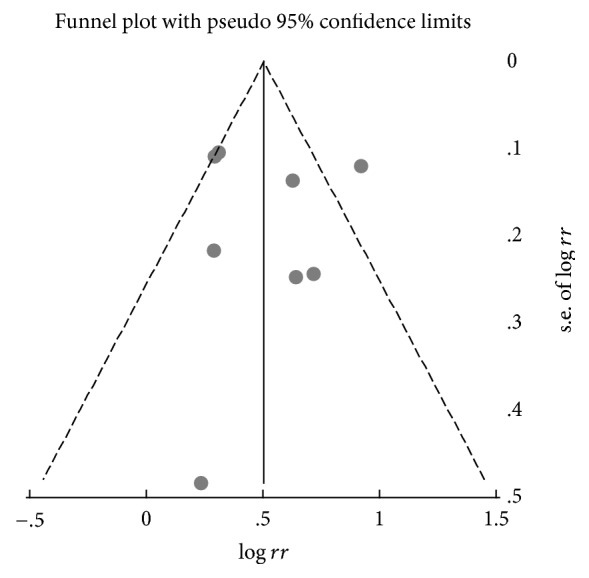
Funnel plot of studies evaluating the association between sleep apnea hypopnea syndrome and atrial fibrillation risk. Begg's regression asymmetry test (*P* = 0.833).

To assess bias across studies, Begg's test with funnel plot asymmetry was used to identify small study effects for the association between SAHS and the risk of AF. We were unable to detect the presence of publication bias in the analyses (*P* = 0.833), indicating a low probability of publication bias (Figure 4).

## 4. Discussion

To our knowledge, this is the first meta-analysis that evaluated the possible effect of sleep apnea hypopnea syndrome and the risk of atrial fibrillation using the results of previous published studies. In this study, we found that the current evidence in the meta-analysis of cohort study suggests that sleep apnea hypopnea syndrome could increase the risk of AF, and there is a dose-response relationship between SAHS severity and rates of incident AF.

SAHS has been shown to contribute to the increased AF burden [21, 22]. Previous studies have reported a strong association between SAHS and AF, with an increased risk of 2- to 4-fold than that of those without SAHS [7]. A prospective cohort study found that patients with SAHS had the risk of AF with OR of 4.02 after adjustment for sex, age, BMI, and coronary heart disease. No dose-response relationship was found between the risk for AF and severe SAHS [7]. On the other hand, another study found that the risk of AF was linearly associated with severity of SAHS; mild SAHS increased AF risk 2.47-fold, and moderate to severe SAHS increased AF risk 5.66-fold [9]. Not all studies, however, have shown a positive association between SAHS and AF. A case-control study failed to confirm the prevalence of obstructive sleep apnea in AF patients and common population (32% versus 29%, *P* = 0.67) [23]. However, the number of subjects in this study was relatively small and the statistical power to detect an association was therefore limited.

There is emerging evidence from animal and human studies that the physiologic changes of sleep apnea, including hypoxia, hypercapnia, and intrathoracic pressure swing, precipitate electrical and structural changes. The exact mechanisms for the association between sleep apnea hypopnea syndrome and atrial fibrillation remain unclear, as the two conditions share many of the same risk factors; however, emerging evidence from animal and human studies indicated that the physiologic changes of sleep apnea including hypoxia, hypercapnia, and activation of sympathetic nervous function may be involved in this process [24]. On the other hand, AF usually reduces cardiac output, which leads to central apnea during sleep, mainly because of the chemoreflex enhancement and prolonged lag to ventilatory response [25]. Previous study reported that obstructive events during sleep promote reductions in the intrathoracic pressure, intermittent hypoxia, and sleep fragmentation [26], which may result in structural cardiac changes, including atrial enlargement and fibrosis [27]. Chronic atrial dilation caused by changes in intrathoracic pressure and surges in blood pressure may facilitate atrial remodeling in SAHS. Besides, SAHS has been reported to increase aorta stiffness that in turn resulted in increased heart afterload and atrial and ventricular remodeling [28, 29]. The increased risk of recurrence of AF has been also reported in patients who performed catheter ablation [30]. Another observational study showed that patients with untreated SAHS have a higher recurrence of AF after catheter ablation. Continuous positive airway pressure treatment was associated with a lower recurrence of AF [31]. SAHS induces repeated episodes of hypoxia which trigger chemoreflex and enhance sympathetic nerve activity, leading to tachycardia and blood pressure elevation. The above changes result in repeated myocardial and subsequently atrial ischemia during sleep, thereby promoting AF [32]. Autonomic nervous system dysfunction may be one of the mechanisms by which SAHS increases the incidence of atrial fibrillation [33]. Collapse of upper airway in patients with OSA may result in increased intrathoracic negative pressure. Upper airway collapse in patients with OSA may cause increased chest negative pressure. Sleep-breathing events can also lead to intermittent apnea, hypoxemia, hypercapnia, and other changes in blood gas, as well as sympathetic activation and subsequent hemodynamic changes, which all contribute to the development of atrial fibrillation [34].

In this meta-analysis, we confirmed that sleep apnea hypopnea syndrome could increase the risk of atrial fibrillation. However, our meta-analysis also has limitations. Although we perform a metaregression analysis to evaluate the influence of variables such as history of cardiovascular disease and BMI on the risk of atrial fibrillation, there was significant heterogeneity among dose-response and subgroup studies; we failed to conduct metaregression analysis in these studies because these variables were always unavailable.

In conclusion, our results indicated that sleep apnea hypopnea syndrome could increase the risk of atrial fibrillation. There is a dose-response relationship between sleep apnea hypopnea syndrome severity and rates of incident atrial fibrillation, and the higher the severity of sleep apnea hypopnea syndrome, the higher the risk of atrial fibrillation.

## Figures and Tables

**Table 1:** Characteristics of studies included in the meta-analysis.

Author	Year	Country	SAHS		Non-SAHS		Research type	Age (year)	Follow-up (year)	SAHS diagnosis	Study quality
			Events	Total	Events	Total					NOS score
Gami et al.	2007	USA	114	2626	19	916	Retrospective cohort study	49.0 ± 14.0	Mean 4.7 years	Polysomnography	8
Khawaja et al.	2013	USA	81	764	1386	17991	Prospective cohort studies	67.7 ± 8.6	Mean 6.9 ± 2.1 years	Questionnaire	7
Chao et al.	2014	China	53	4082	3970	575439	Longitudinal cohort study	38.9 ± 13.1	Mean 9.2 ± 2.0 years	Polysomnography	8
Kwon et al.	2015	USA	72	1341	28	707	Multisite cohort study	68.4 ± 9.2	2 years and 4 months	Polysomnography	7
Cadby et al.	2015	Australia	374	4352	81	2489	Consecutive cohort study	48.3 ± 12.5	Median 11.9 years	Polysomnography	8
Lin et al.	2015	USA	16	165	196	4018	Prospectively longitudinal cohort study	61.3 ± 9.6	Mean 8.5 years	Questionnaire	6
May et al.	2016	USA	4	29	53	500	Prospective cohort studies	75.0 ± 5.0	Mean 6.5 ± 0.7 years	Polysomnography	7
Tung et al.	2017	USA	196	1440	142	1472	Prospective cohort studies	62.8 ± 11.2	Average 5.3 years	Polysomnography	7

SAHS: sleep apnea hypopnea syndrome; NOS: Newcastle-Ottawa Scale.

**Table 2:** Metaregression (inverse variance weights, *n* = 9).

Var.	Coeff.	Std. Err.	*P* value	95% Conf. Interval
Year	−0.0216	0.0402	0.61	(−0.1200, 0.0767)
Age	−0.0174	0.0068	0.045	(−0.0342, −0.0005)
Gender	2.00*e* − 04	0.5342	1	(−1.3070, 1.3076)
Sample size	2.08*e* − 07	5.20*e* − 07	0.702	(−1.06*e* − 06, 1.48*e* − 06)
Follow-up duration	0.0721	0.0223	0.018	(0.017, 0.1269)
NOS score	0.2493	0.131	0.106	(−0.0712, 0.5698)
SAHS diagnosis	−0.1417	0.234	0.567	(−0.7145, 0.4310)
Country	0.4266	0.126	0.015	(0.1182, 0.7350)

NOS: Newcastle-Ottawa Scale; SAHS: sleep apnea hypopnea syndrome.
